# Lack of spatial segregation in the representation of pheromones and kairomones in the mouse medial amygdala

**DOI:** 10.3389/fnins.2015.00283

**Published:** 2015-08-11

**Authors:** Vinicius M. A. Carvalho, Thiago S. Nakahara, Leonardo M. Cardozo, Mateus A. A. Souza, Antonio P. Camargo, Guilherme Z. Trintinalia, Eliana Ferraz, Fabio Papes

**Affiliations:** ^1^Department of Genetics and Evolution, Institute of Biology, University of CampinasCampinas, Brazil; ^2^Graduate Program in Genetics and Molecular Biology, Institute of Biology, University of CampinasCampinas, Brazil; ^3^Undergraduate Program in the Biological Sciences, Institute of Biology, University of CampinasCampinas, Brazil; ^4^Campinas Municipal ZooCampinas, Brazil

**Keywords:** olfaction, vomeronasal organ, amygdala, higher-order representation, pheromone, kairomone

## Abstract

The nervous system is organized to detect, internally represent and process sensory information to generate appropriate behaviors. Despite the crucial importance of odors that elicit instinctive behaviors, such as pheromones and kairomones, their neural representation remains little characterized in the mammalian brain. Here we used expression of the immediate early gene product c-Fos as a marker of neuronal activity to find that a wide range of pheromones and kairomones produces activation in the medial nucleus of the amygdala, a brain area anatomically connected with the olfactory sensory organs. We see that activity in this nucleus depends on vomeronasal organ input, and that distinct vomeronasal stimuli activate a dispersed ensemble of cells, without any apparent spatial segregation. This activity pattern does not reflect the chemical category of the stimuli, their valence or the induced behaviors. These findings will help build a complete understanding of how odor information is processed in the brain to generate instinctive behaviors.

## Introduction

In mammals, sensory information is detected by specialized sensory cells at the periphery and is then sent to the brain, where it must be systematically represented by coherent patterns of neural activity (Luo and Flanagan, 2007). Though still poorly understood, it is assumed that these patterns of activity are interpreted, resulting in output behaviors or endocrine changes.

In the visual and somatosensory systems, the discrimination of stimuli located in different positions in the sensed environment is achieved by their representation in topographic (or continuous) maps in the brain. For example, neighboring activated retina cells, representing adjacent sources of light in the visual field, send projections to neighboring neurons in the thalamus and visual cortices, such that the ordering of sensory stimuli in the external world is represented by ordered maps of neural activity in the brain (Luo and Flanagan, 2007). In contrast, gustatory information is represented in a non-continuous, or discrete, fashion, where different taste qualities, such as sweet, bitter, umami, and salty, resulting from the detection of the corresponding tastants in the upper digestive system, are each represented by cohorts of activated neurons in discrete sub-areas of the primary taste cortex in the brain (Chen et al., 2011).

Less is known about how olfactory information is internally represented in the brain. The olfactory system is specialized in the sensory detection of a variety of chemical stimuli, which indicate the presence and quality of food, potential mates, competitors, and dangers in the environment (reviewed in Munger et al., 2009). Though the central representation of regular volatile odorant stimuli in the brain has been recently investigated (Stettler and Axel, 2009; Sosulski et al., 2011), very little is known about how odors able to elicit instinctive responses, such as pheromones and kairomones, are internally represented by coherent activity in olfactory brain areas. Pheromones (released by an individual and detected by the same species) and kairomones (released by an individual and detected by another species) are chemosignals that mediate a range of instinctive behavioral responses, including aggression (Chamero et al., 2007), mating, gender discrimination (Stowers et al., 2002; Kimchi et al., 2007), and fear (Papes et al., 2010). Since these cues crucially regulate the interactions between individuals, the study of their neural representation is central to understanding how the brain controls animal behavior, indirectly impacting life cycle, natural history, and evolution.

The vomeronasal organ (VNO), an olfactory structure in the nasal cavity, has been implicated in the detection of some pheromones and kairomones (Munger et al., 2009). Substances detected by VNO sensory neurons include urine-derived small organic molecules (Sam et al., 2001; Trinh and Storm, 2003), sulfated steroids (Nodari et al., 2008; Isogai et al., 2011), MHC peptides (Leinders-Zufall et al., 2004), peptides in the ESP family (Haga et al., 2010; Ferrero et al., 2013) and conspecific and heterospecific small proteins in the Major Urinary Protein family (Mup) (Chamero et al., 2007; Papes et al., 2010; Kaur et al., 2014; Dey et al., 2015), which mediate the instinctive behaviors mentioned above.

Vomeronasal sensory neurons connect directly with the accessory olfactory bulb (AOB) in the brain via the vomeronasal nerve (Wagner et al., 2006). In turn, the AOB is anatomically connected to several brain areas, including nuclei in the amygdala, such as the posteromedial cortical nucleus of the amygdala (PMCO) and the medial nucleus of the amygdala (MeA) (Canteras et al., 1995; Petrovich et al., 2001).

Pheromones and kairomones lead to behaviors instinctively, and are thus believed to be processed by hard-wired brain circuits. Moreover, the behaviors mediated by the VNO are stereotypical (conserved between individuals), and therefore we assumed that pheromone/kairomone information is represented by coherent activity along those circuits. However, the organizing principles behind such brain activity remain poorly characterized. Prior studies have shown that activity in the AOB reflects sensory input from the VNO (Wagner et al., 2006). In contrast, it is thought that other regions are organized to reflect behavioral output. For example, cat-odorized pieces of collar, which induce defensive behaviors in mice, lead to prominent neural activity in the ventral part of the MeA (Dielenberg et al., 2001; Choi et al., 2005), while female mouse odors, which trigger reproductive responses, activate the dorsal MeA (Fernandez-Fewell and Meredith, 1994; Kollack-Walker and Newman, 1997; Choi et al., 2005). These findings led to the idea that the MeA is divided into dorsal and ventral sub-areas, activated by predatory and social stimuli, respectively, composing distinct pathways involved in defensive or reproductive behaviors (Swanson, 2000; Canteras, 2002; Choi et al., 2005; Lin et al., 2011; Silva et al., 2013). However, these notions arose from the use of a very limited set of olfactory stimuli and no systematic comparison has been made among a range of stimuli eliciting various behavioral outputs, to understand how and where sensory information represented along the circuit initiated at the VNO transitions to the generation of behavior by downstream brain areas.

Here we investigated and compared how different olfactory stimuli, each able to induce instinctive behaviors following detection mediated by the VNO, generate activity in the amygdala. Because the MeA is one of the first higher-order brain regions to receive information collected by the VNO, we decided to study the patterns of activity in this nucleus, in groups of animals exposed to distinct types of olfactory cues. In order to create a detailed view of how MeA activity is organized, we chose a comprehensive approach, where activity was evaluated in mice exposed to a wide range of pheromones and kairomones. First, we analyzed activity in the AOB, as a confirmation that the VNO has been activated by each employed stimulus. We then showed that a large set of intra- and interspecies signals leads to activation in the MeA, in a VNO-dependent manner. We also obtained evidence that neural activity in this nucleus is not organized to reflect the valence or behavioral consequences of each detected stimulus, as previously thought. Instead, we found a lack of any discernible spatial map in the MeA, where each stimulus activates dispersed ensembles of neurons and distinct stimuli activate intermingled sets of cells. Therefore, it is unlikely that the amygdala contains a spatial map to represent different pheromones and kairomones. This knowledge will be key to comprehending how the brain's limbic system represents external olfactory information important for the survival of the individual and the species.

## Materials and methods

### Animals

Animals were 8 weeks old male mice (females, where indicated). In some experiments, MeA activity was evaluated in animals where VNO neurons were genetically ablated by a null mutation in the gene *TrpC2*, coding for the primary vomeronasal sensory transduction channel (Stowers et al., 2002). TrpC2^+/+^ and TrpC2^−/−^ littermates were obtained from heterozygous mating couples, which were produced by backcrossing the TrpC2^−/−^ knockout line (Stowers et al., 2002) into the C57BL/6J background for at least 10 generations (Papes et al., 2010). Animals had no previous exposure to odors from other animal species, and subjects exposed to conspecific chemosignals were kept individually caged for at least 4 days. All subjects were exposed to odor, monitored for behavior, and subsequently processed for immunostaining or *in situ* hybridization, ensuring that the cellular responses and behaviors were analyzed from the same individuals and no animals were re-used. This study was carried out in accordance with Animal Protocol no. 1883-1, approved on June 2009 by the Institute of Biology's Institutional Animal Care and Use Committee (Committee for Ethics in Animal Use in Research), at the University of Campinas. This protocol follows the guidelines established by the National Council for Animal Experimentation Control (CONCEA-Brazil).

### Stimuli

Mouse subjects were separately exposed to a wide range of intra- and interspecies odor stimuli, known to induce biologically relevant instinctive behaviors, such as odors from adult female and male mice, juveniles, odors from other mouse strains (BALB/c and 128S/J), and numerous species that are natural or occasional mouse predators (rat, domestic cat, leopard cat, Cougar mountain lion, African lion, several snake species, great horned owl, caracara hawk, and tarantula spider). In principle, olfactory stimuli should ideally be presented in the same form and amount, such as equal volumes of scented bedding. However, because home cage bedding may contain noxious compounds from feces or urine, we decided to use gauze scented with bodily secretions (urine, skin secretions) or bodily shedding (feathers, fur, skin) whenever possible. Table S1 presents a complete list of stimuli and collection methods. Cat-scented gauze was obtained by rubbing a medical gauze against the fur of a domestic cat, particularly around the neck region (Papes et al., 2010). Fifty milliliters of scented bedding (fine wood chips) were used as odors from leopard cat, mountain lion, African lion, tarantula spider, rat, and male and female mice. Alternatively, rat urine was used in some experiments by placing 1 ml of urine on pieces of medical gauze. For all stimuli deposited on gauzes, the gauze was unscented in a desiccator under vacuum overnight before adding the stimulus. For each snake species, we used 1 g of stimulus (around four 5 × 5 cm pieces of shed skin). Avian predator stimuli (hawk and owl) were 1 g of feathers, cut into small pieces. All stimuli (solid or liquid deposited on gauze) were attached to “binder clips” to visually confirm their position and prevent the spreading of stimuli in the cage. Control mice were exposed to unscented control odors, as indicated in Table S2. Some of the aforementioned odors were presented in different forms and most are composed of complex mixtures of largely uncharacterized ligands, some of which may also activate other sensory systems. Thus, additional experiments were performed to determine with certainty that activity in the MeA was due to pheromones and kairomones, using Mup proteins as pure ligands. These Mups are contained within the corresponding complex mixtures and could be presented in comparable amounts. For Mup experiments (**Figure 3**), gauze was scented with 10 mg of recombinant protein as fusion with Maltose-Binding Protein (MBP), and gauze scented with MBP alone was used as control.

### Behavioral assays

For defensive avoidance behavior, individually caged mice were habituated for 2 days in the dark in the procedure room and assayed on day 3. The amounts used for each stimulus are indicated in Table S1. In all cases, the animal was placed in the procedure room on day 3 and the stimulus was deposited on the side opposite to the air inlet. Mice were exposed and filmed for 30 min in the dark. Movies were scored blindly for avoidance time, following previously published protocol (Papes et al., 2010). Avoidance behavior was defined as the amount of time animals spent more than 20 cm away from the stimulus. To compare avoidance times where needed, ANOVA was applied, followed by Tukey-Kramer Honest Significantly Different (HSD) *post-hoc* analysis. Other types of defensive behaviors were also assayed (not shown), including freezing and risk assessment (Papes et al., 2010); in the latter, the animal approaches the stimulus with an extended body posture and arched back, a behavior seen for all predatory stimuli; during these episodes, the animals closely approached the stimulus in the first 5 min of stimulation; close contact was eventually seen after 25 min from the onset of stimulation, at which time the defensive behaviors became of progressively lower magnitude; eventual licking and biting the gauze was observed at the end of the exposure sessions, but preliminary experiments determined that physical contact with the stimulus is not necessary to trigger the aversive behaviors or brain activity, suggesting that the stimuli employed (even non-volatile Mup proteins) accessed the VNO lumen as airborne aerosol particles. For aggressive behavior assays, C57BL/6J male mice (8–12 weeks old) were isolated for 1 week and then exposed to castrated adult mice swabbed with 40μl of test solution (male mouse urine or equivalent amounts of rat or rabbit urine) for 10 min in their home cages. Tests were videotaped and analyzed to measure total duration of aggressive contact (biting, wrestling, and kicking). One round of urine and no-urine controls were performed with each resident mouse before and after sample testing. For reproductive behavior assay, the resident was a male mouse, prepared the same way as in the aggressive behavior assays. Subjects were exposed to 8 weeks old sexually naive, receptive females. Each assay ran for 15 min and the filmed behaviors were scored for mounting time (not shown).

### Recombinant Mup protein expression

The cDNA for rat major urinary protein, Mup13 (Logan et al., 2008), was amplified by PCR from a Sprague-Dawley liver sample using oligonucleotides 5′ ATCGGATCCCATGCAGAAGAAGCTAGTTCCACAAGAG 3′ and 5′ ATCAAGCTTTCATCCTC GGGCCTGGAGACAG 3′. The amplicon was cloned into pMAL-c2x bacterial expression vector (New England Biolabs) into *Bam*HI and *Eco*RI restriction sites, and expressed as a fusion protein with MBP, following the manufacturer's recommendations. Protein was eluted from an amylose affinity resin using maltose and then exchanged into 1x PBS using a YM10 column (Millipore) prior to exposures. Recombinant MBP was used as a control. The same procedure was applied for production of the mouse Mups (nomenclature following Logan et al., 2008), and for the production of recombinant cat Mup, except that the corresponding cDNA was synthesized *in vitro* based on the published sequence of Fel-d-4 (cat Mup; GenBank accession number NM_001009233) (Smith et al., 2004).

### c-Fos immunostaining

For brain activity analyses, the expression of the surrogate marker of neuronal activity c-Fos was assayed by immunostaining. Inspection of neural activity by electrophysiological methods is very difficult for the MeA, due to its small size and deep location, but direct recording of activity was shown to be highly correlated with expression of the immediate early gene *c-Fos* in previous reports (Lin et al., 2011).

Each animal was individually caged and habituated to the procedure room where the exposures were conducted for 2 h on 2 consecutive days, in the dark. On the third day, each cage was brought to the procedure room and the stimulus was introduced in the animal's home cage on the side opposite to the air inlet. Each animal was exposed for 30 min, and the stimulus was removed from the cage at the end of the session, after which the subject remained in the dark without further stimulation for an additional period of 60 min; the animal was then quickly euthanized and dissected to remove the brain. Brains were fixed overnight in 4% paraformaldehyde, equilibrated in 20% sucrose/1x PBS and sectioned on a Leica 1000S vibrating-blade microtome. Fifty micrometer coronal sections were collected for the entire brain, and suitable sections were chosen for subsequent c-Fos immunostaining based on comparisons to a reference brain atlas (Paxinos and Franklin, 2004). Sections were blocked as free-floating sections for 1 h with 1% blocking reagent (Invitrogen), pre-incubated in 1% BSA/1x PBS/0.3% Triton X-100, followed by incubation with the anti-c-Fos primary antibody (rabbit polyclonal; Ab5; Millipore) diluted 1:1500 in 1% BSA/1x PBS/0.3% Triton X-100 for 36 h at 4°C under gentle agitation. Sections were washed three times in 1xPBS/0.1% Triton X-100, 15 min each, and incubated for 3 h at room temperature with Alexa 488-conjugated goat anti-rabbit secondary antibody (Invitrogen) diluted 1:500 in 1% BSA/1x PBS/0.3% Triton X-100. After two washes in 1x PBS/0.1% Triton X-100, 15 min each, sections were counterstained with To-Pro-3 nuclear stain (Invitrogen) diluted 1:1000 in 1x PBS, washed twice in 1x PBS, 15 min each, and mounted onto glass microscope slides with ProLong Gold (Invitrogen). Dry mounted sections were imaged on a Leica TCS SP5 confocal fluorescence microscope. The number of c-Fos positive nuclei was counted blindly for each individual. After image acquisition, the nuclear stain channel was computationally false-colored as purple, to facilitate contrast with the green fluorescence channel (c-Fos).

### RNA *in situ* hybridization

For VNO activity analyses, expression of the surrogate marker of vomeronasal neuron activity *Egr1* (Isogai et al., 2011) was used. Animals were exposed to stimulus for 45 min, sacrificed and the VNOs immediately collected, immersed in 4% paraformaldehyde fixative overnight, equilibrated in sucrose and sectioned on a cryostat (Leica) to produce 16 μm transversal sections. Slides were air-dried for 10 min, followed by fixation with 4% paraformaldehyde for 20 min, and treatment with 0.1 M HCl for 10 min, with 0.1% H_2_O_2_ for 30 min and with 250 mL of 0.1 M triethanolamine (pH 8.0) containing 1 mL of acetic anhydride for 10 min, with gentle stirring. Slides were always washed twice in 1x PBS between incubations. Hybridization was then performed with DNP (1 μg/mL) or DIG (600 ng/mL) labeled cRNA probes (Isogai et al., 2011) at 58°C in hybridization solution (50% formamide, 10% dextran sulfate, 600 mM NaCl, 200 μg/ml yeast tRNA, 0.25% SDS, 10 mM Tris-HCl pH8.0, 1x Denhardt's solution, 1 mM EDTA pH 8.0) for 16 h. Slides were washed once in 2x SSC, once in 0.2x SSC and once in 0.1x SSC at 60°C (30, 20, and 20 min, respectively), followed by a quick incubation in 0.1x SSC at room temperature. Slides were then permeabilized in 1x PBS, 0.1% Tween-20 for 10 min, and washed twice in TN buffer (100 mM Tris-HCl pH 7.5, 150 mM NaCl) for 5 min at room temperature, followed by blocking in TNB buffer [100 mM Tris-HCl pH 7.5, 150 mM NaCl, 0.05% blocking reagent (Perkin Elmer)], and incubation with rabbit anti-DNP (Invitrogen) primary antibody diluted 1:600 in TNB buffer overnight at 4°C. Signal development proceeded with the tyramide signal amplification kit (Perkin Elmer), following the manufacturer's instructions. Briefly, slides were incubated in tyramide-biotin [1:50 in amplification diluent with 0.0015% H_2_O_2_ (Perkin Elmer)] for 15 min, followed by incubation in streptavidin-HRP (1:100 in TNB) for 1 h, followed by incubation in tyramide-Alexa Fluor 546 [1:100 in amplification diluent (Life Technologies) with 0.0015% H_2_O_2_] for 15 min. Prior to each incubation, slides were washed 6 times with TNT buffer for 5 min under mild agitation. Sections were then treated with 3% H_2_O_2_ in 1x PBS for 1 h to block peroxidases from the first signal development. Slides were then blocked in TNB for 90 min, followed by incubation overnight at 4°C with anti-DIG-POD (Roche) diluted in TNB (1:400). Signal development was performed using tyramide-Alexa Fluor 488 dye (Invitrogen). Samples were counter-stained with To-Pro 3 nuclear stain (Invitrogen) diluted 1:1000 in 1x PBS, washed twice in 1x PBS and mounted with ProLong Gold (Invitrogen). Dry mounted sections were imaged on a Leica TCS SP5 confocal fluorescence microscope.

### Dual immunostaining/*in situ* hybridization

We developed a dual staining protocol to parse out, in the same animal, the sets of active neurons related to two sequential exposure events separated by a period with no stimulation. In brief, mice were first exposed to a stimulus, then transferred back to their own soiled home cages for some time, followed by a second exposure to stimulus. Brains were then subjected to a dual immunostaining/*in situ* hybridization protocol to detect immature nuclear *c-Fos* mRNA derived from the second exposure period, concomitant with the detection of nuclear c-Fos protein resulting from the first exposure period. Specifically, animals were individually caged in Cage #1 and habituated in the dark for 90 min per day on the previous 2 days before the exposure. The habituation protocol guarantees that the animals are exposed to olfactory stimuli in a context relevant to the generation of behaviors. On the exposure day, half of the soiled bedding from the animal's cage was transferred to another cage (Cage #2). The first exposure to olfactory stimulus was performed in Cage #1 for 20 min, and the animals were then transferred to Cage #2 for 60 min without olfactory stimulation. They were then transferred back to Cage #1 for a second period of olfactory stimulation for 20 min (same or different stimulus). All of these steps were conducted in the dark. At the end of the exposures, animals were anesthetized with ketamine/xylazine, and quickly perfused with 4% paraformaldehyde fixative. Brains were further fixed overnight with RNAse-free fixative and equilibrated in RNase-free 20% sucrose. Forty micrometer sections were collected on a VT100S vibratome (Leica) in 1x PBS-DEPC. Chosen sections encompassing the MeA were subjected to a new method for the combined detection of *c-Fos* mRNA and protein, as follows. *Pre-treatment of sections and probe hybridization:* Free-floating sections were fixed with 4% paraformaldehyde for 20 min, permeabilized in 0.2 M HCl/H_2_O-DEPC for 10 min, incubated in 0.1% H_2_O_2_/1x PBS-DEPC to inactivate endogenous peroxidases for 30 min and acetylated in 0.1 M Triethanolamine-HCl pH 8.0 with acetic anhydride for 10 min. Sections were then incubated in hybridization solution containing 400 ng/mL of each of two 1 kb digoxigenin-labeled cRNA probes in a 5x SSC humidified chamber for 16 h, at 58–60°C. Probes correspond to two fragments of the *c-Fos* mRNA (fragments were obtained by reverse transcription-PCR using the following oligonucleotides: Probe 1: 5′ CAGCGAGCAA CTGAGAAGAC 3′ and 5′ GCTGCATAGAAG GAACCGGAC 3′; Probe 2: 5′ GGAGCCAGTCA AGAGCATCAG 3′ and 5′ AATGAACATTG ACGCTGAAGGAC 3′). Hybridization solution also contained 50% deionized formamide, 600 mM NaCl, 200 μg/mL yeast tRNA, 0.25% SDS, 10 mM Tris-HCl pH 8.0, 1x Denhardt's solution, 1 mM EDTA pH 8.0 and 10% dextran sulfate. *Washes and antibody incubation:* Sections were washed in 2x, 0.2x, and 0.1x SSC solutions (20 min each), permeabilized in 0.1% Tween 20/1x PBS and blocked in 100 mM Tris-HCl/150 mM NaCl/0.5% Blocking Reagent (Perkin Elmer). Next, sections were incubated with alkaline phosphatase-conjugated anti-digoxigenin antibody (Roche; 1:400) for 2 nights, at 4°C. *Signal amplification and immunostaining:* Sections were incubated in tyramide-biotin (Perkin Elmer; 1:50) in amplification diluent containing 0.0015% H_2_O_2_, then incubated with horseradish peroxidase-conjugated streptavidin (Perkin Elmer; 1:100) in 100 mM Tris-HCl/150 mM NaCl/0.5% Blocking Reagent, and finally incubated in Alexa Fluor 546-tyramide (Invitrogen; 1:100) in amplification diluent containing 0.0015% H_2_O_2_. Peroxidase from the first signal was inactivated by treatment with 3% hydrogen peroxide for 30 min and 0.1 HCl for 10 min, prior to blocking and anti-c-Fos primary antibody (Ab5; Millipore) incubation in regular c-Fos immunostaining as detailed before. Nuclear counterstaining was performed with To-Pro-3 (Invitrogen; 1:1000) and sections were mounted on glass slides with ProLong Gold anti-fade reagent (Invitrogen) and imaged on a Leica TCS SP5 confocal microscope.

Since time elapsing between the two episodes of stimulation is sufficiently long in our protocol, this strategy enabled us to parse out the activation profiles derived from both stimulations with great precision, such that c-Fos mRNA is indicative of activation during the last exposure and c-Fos protein is indicative of the first exposure (**Figures 9D–G**). This method was based on the catFISH procedure (Guzowski et al., 1999, 2001; Lin et al., 2011). However, our method enables better resolution in the assignment of brain activity resulting from each of two consecutive olfactory stimulations, because the two exposure episodes are separated by a longer time period (1 h). Moreover, because the *c-Fos* gene encodes a transcription factor, the co-detection of its mRNA and protein inside the same subcellular compartment (nucleus) makes it unequivocal to determine if the cell produced mRNA, protein or both.

### RNA probe design and validation

For the design of cRNA probes to V2R vomeronasal receptors, we investigated whether different receptor genes harbor specific regions anywhere in the coding or non-coding regions. However, nucleotide and protein similarities among the members of each clade were found to be very high (>80%), though members from different clades usually share less than 70% nucleotide sequence identity. These similarity levels are constant throughout the entire gene sequence, including exons, introns, and untranslated regions. Unlike previously published reports (Isogai et al., 2011), one could not obtain probes that permit safe discrimination between cells expressing different receptors in the same clade, even under highly stringent hybridization conditions. For each clade, we chose probes based on one or two receptors, and each probe exhibited 80% minimum nucleotide similarity with other receptor members in the same clade, but 72% maximum allowed similarity with receptors in other clades. The rate of co-labeling of cells with two differently labeled probes in the same clade was high in prior validation experiments (usually >90% concordance). Each cRNA probe was produced with rNTPs labeled with haptens DNP (Egr1) and/or DIG (V2R) (Roche) from 1 kb suitable fragments cloned into pGEM-T-Easy vector (Promega), using SP6 or T7 RNA polymerases (Roche). VNO neurons in the basal zone co-express receptors in the V2R A/B/D families plus one receptor in the V2R C family (Silvotti et al., 2007). Since C family members are widely expressed in these neurons, we did not include probes for them in our investigation of the molecular identity of neurons activated by each olfactory stimulus.

### Statistical analyses

Statistical analyses were performed using R and Stat packages, and XLSTAT add-on in Excel. For comparing mean behavioral output measurements and numbers of c-Fos positive cells in each brain region, we applied one-way Analysis of Variance (ANOVA), followed by Tukey-Kramer HSD *post-hoc* analysis. In each case, *P*-values indicate the probability that the null hypothesis (the means are equal or were drawn from like populations) is true. *P* > 0.05 led to rejection of the null hypothesis in all tests. For comparing distributions of active cells in the MeA for two stimulus groups in **Figure 11**, we counted cells along the dorsal-ventral axis of the MeA, the position and direction of which were estimated taking the third ventricle in the hypothalamus as a proxy. The axis ventral limit (the origin in all graphs in **Figure 11**) was defined as the most ventral pixel in the MeA in each image. The axis dorsal limit was defined as the point 600 μm from the origin along the dorsal-ventral axis. Data collected at bregma –1.46 mm from all animals in each group being compared were plotted in scale intervals of 60 μm (total of 10 intervals plotted in bar graphs in **Figure 11**) along the dorsal-ventral MeA axis. Comparisons between distributions for any two groups were performed with the Kolmogorov-Smirnov distribution comparison test (KS test), applied on cumulative ranks for the intervals mentioned above. In this case, *P*-values indicate the probability that the null hypothesis (the distributions are equal or were drawn from like populations) is true.

## Results

### A wide range of intra- and interspecies chemosignals detected by the VNO activate the medial nucleus of the amygdala

**Figure 1:**
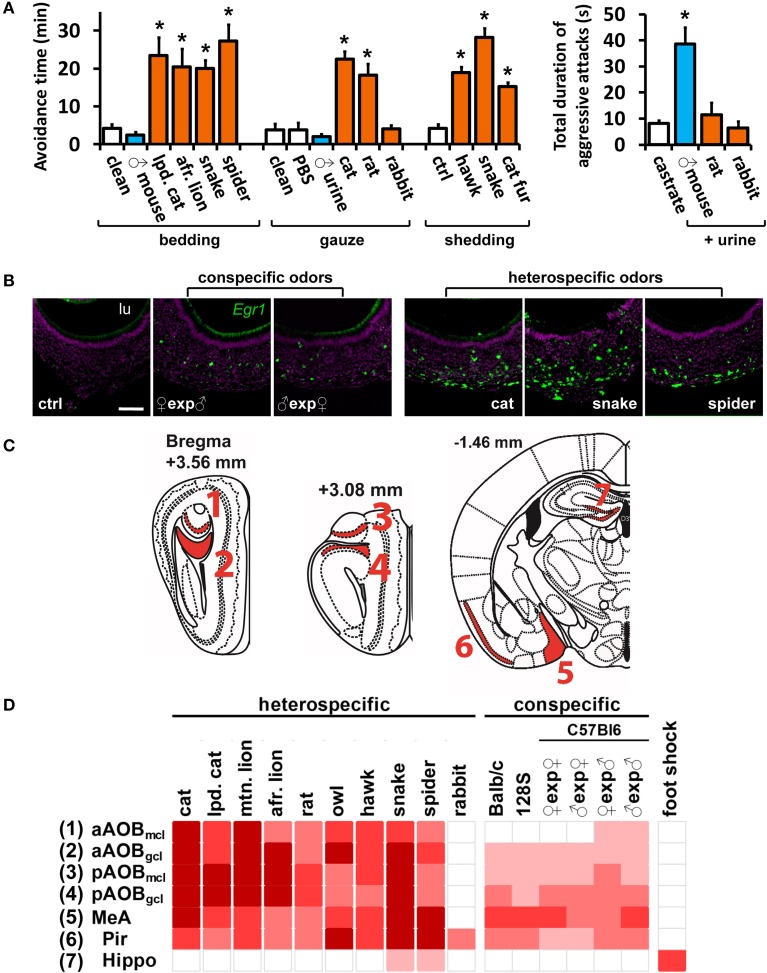
**A wide range of olfactory stimuli activates the medial nucleus of the amygdala. (A)** Increased avoidance defensive behavior (left) after exposure to various heterospecific mammalian and non-mammalian predator odors. Some heterospecific (rabbit) and conspecific stimuli do not induce behavior. In contrast, inter-male aggression (right) is elicited by conspecific, but not by heterospecific, odors. Heterospecific stimuli are shown in orange and conspecific ones in blue, grouped according to presentation form (bedding, gauze or bodily shedding). Mean ± s.e.m. ^*^p < 0.01; ANOVA followed by Tukey-Kramer HSD *post-hoc* analysis against respective control (white bars). See Tables S1 and S2 for a list of stimuli and controls. **(B)** Exposure of male mice to conspecific or heterospecific stimuli activates VNO neurons, as evidenced by *in situ* hybridization to immediate early gene *Egr1*. Control (ctrl) odor is PBS-soaked gauze for liquid stimuli (similar number of *Egr1*-positive cells were found for other controls). Scale bar represents 100 μm. lu, VNO lumen. Purple labeling, nuclear stain; green, *Egr1 in situ* hybridization signal. **(C)** Representation of coronal sections through the mouse brain at the indicated bregma values, showing the location of analyzed brain nuclei in **(C)**. 3V, third ventricle; Aq, cerebral aqueduct. **(D)** Heat map representing the activation of the medial nucleus of the amygdala (MeA) and accessory olfactory bulb (AOB) sub-regions, in animals exposed to various heterospecific and conspecific olfactory stimuli. As a control, activity in the piriform cortex (Pir), which does not receive major vomeronasal system inputs, is also shown, as well as the hippocampus (hippo), the activity of which is not directly related to olfaction and was used as control. Bright red indicates the largest number of observed c-Fos-positive cells per unit area for each nucleus. White indicates activation comparable to control level. See Table S2 for numbers of c-Fos-expressing cells and a key to heat map colors in each area, for each odor and respective controls. A white gap separating two columns indicates that the corresponding stimuli were presented in different forms and should not be compared. ♀exp♂ denotes a female mouse exposed to male odors. *n* = 6–26. gcl, granule cell layer of the AOB; mcl, mitral cell layer. n.d., not determined.

In order to investigate in detail the patterns of activity in the MeA, we separately exposed C57BL/6 mice to the various intra- and interspecies olfactory stimuli listed in Table S1 (see also Materials and Methods). For all stimuli, the amounts employed induced similarly potent behavioral responses in mouse subjects (Figure 1A; see also Chamero et al., 2007; Papes et al., 2010; Isogai et al., 2011). Interestingly, several heterospecific odors were shown to trigger defensive behaviors (Figure 1A). Other odors (such as same-strain mouse odors) were unable to elicit defensive behaviors, and instead induced aggressive or reproductive responses (Figure 1A).

With this protocol, increases in VNO and brain activity for each stimulus could be evaluated by comparison with appropriate unscented controls (listed in Table S2). Most of the stimuli activated a large number of VNO sensory neurons (Figure 1B), as judged by the expression of the marker of VNO activity *Egr1* (Isogai et al., 2011). Some stimuli were presented in different forms (Table S1) and therefore cannot be compared, but we noticed a tendency for intraspecific signals to activate a smaller subpopulation of VNO cells than interspecific stimuli, such as predator odors (Figure 1B).

**Figure 2:**
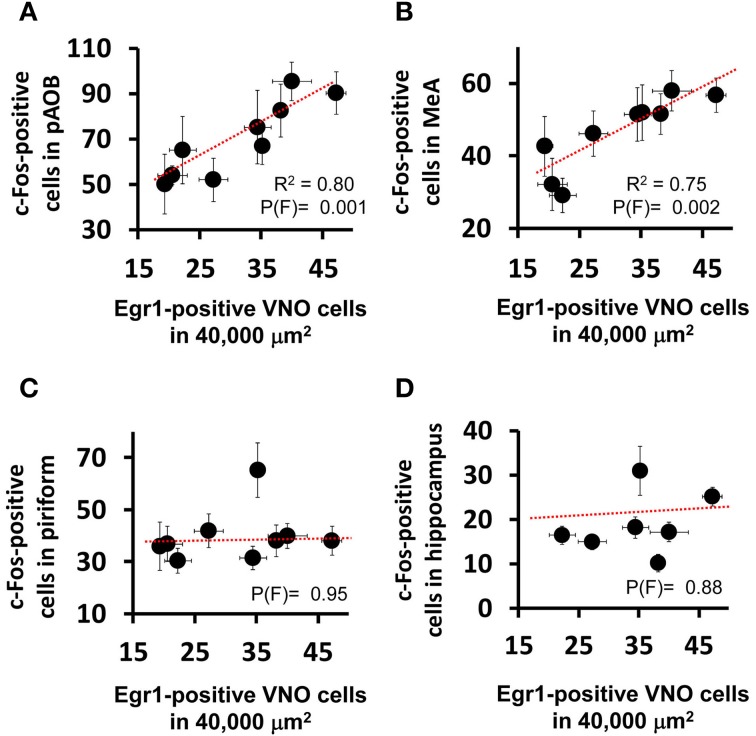
**Activity in the medial nucleus of the amygdala is correlated with vomeronasal sensory detection activity**. Scatter plots comparing the activation level in the VNO (judged by the number of nuclei expressing the immediate early gene *Egr1* in a 40,000 μm^2^ area of sensory epithelium) and the activation level seen in **(A)** AOB (posterior aspect) and **(B)** MeA, as judged by the number of c-Fos positive cells in each brain region per unit area (as denoted in Table S2). Each dot represents a different stimulus. The *R*^2^ and probability values for the F statistics are given in each panel, indicating the amount of variance explained by the linear regression model (dashed red line) and the likelihood that the data fit the model, respectively. Note the positive correlation between AOB and MeA activation and VNO activation, but absence of correlation for the piriform cortex **(C)**, the activation of which reflects events in the main olfactory epithelium, not the VNO. **(D)** The hippocampus is used as a control region not directly affected by either the main olfactory or vomeronasal system activation.

**Figure 3:**
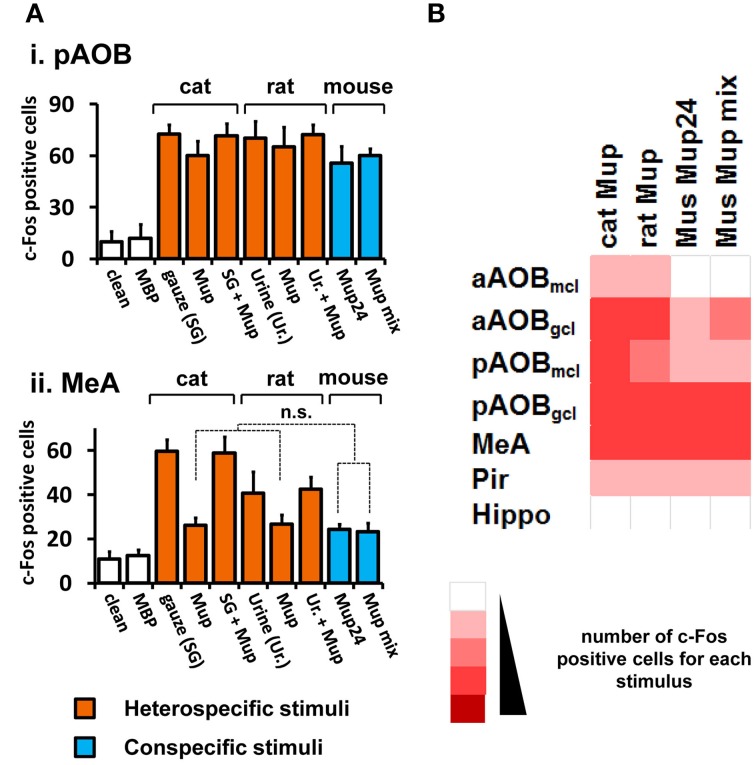
**Activity induced by pure VNO stimuli in the medial nucleus of the amygdala and control brain areas. (A)** Comparison among activity in the AOB (i) and MeA (ii) in animals separately exposed to purified ligands presented in the same amount (cat, rat or mouse Mups). Mup24 or a mixture of Mup24, Mup3, Mup8, and Mup25 (Mup mix) were used at the same total amount. The number of c-Fos expressing cells is also shown for corresponding native stimuli, which are cat-scented gauze (SG) and rat urine (Ur.). Heterospecific stimuli are indicated in orange and conspecific ones in blue. White bars represent unscented controls (MBP-soaked or clean gauze for liquid or solid stimuli, respectively). *n* = 8–12. n.s., no statistically significant difference. **(B)** Heat map representing the activation of the AOB, MeA and control areas (same as in Figure 1) in animals exposed to purified heterospecific and conspecific olfactory stimuli. Abbreviations and color grading are the same as in Figure 1. See Table S2 for numbers of c-Fos expressing cells.

Next, we analyzed whether VNO activation was accompanied by activity in the AOB and MeA, known to have anatomical connections (direct or indirect) with olfactory sensory organs (Figures 1C,D). In animals exposed to heterospecific or conspecific odors, induction of the marker of neuronal activity c-Fos was strong in the AOB (Figures 1D, 2A, 3Ai, 4; see also Papes et al., 2010) and MeA (Figure 1D and Table S2). Rabbit urine, which does not induce defensive or aggressive behaviors in mice, did not activate these nuclei, nor did generally noxious stimuli such as foot shock (Figure 1D and Table S2).

**Figure 4:**
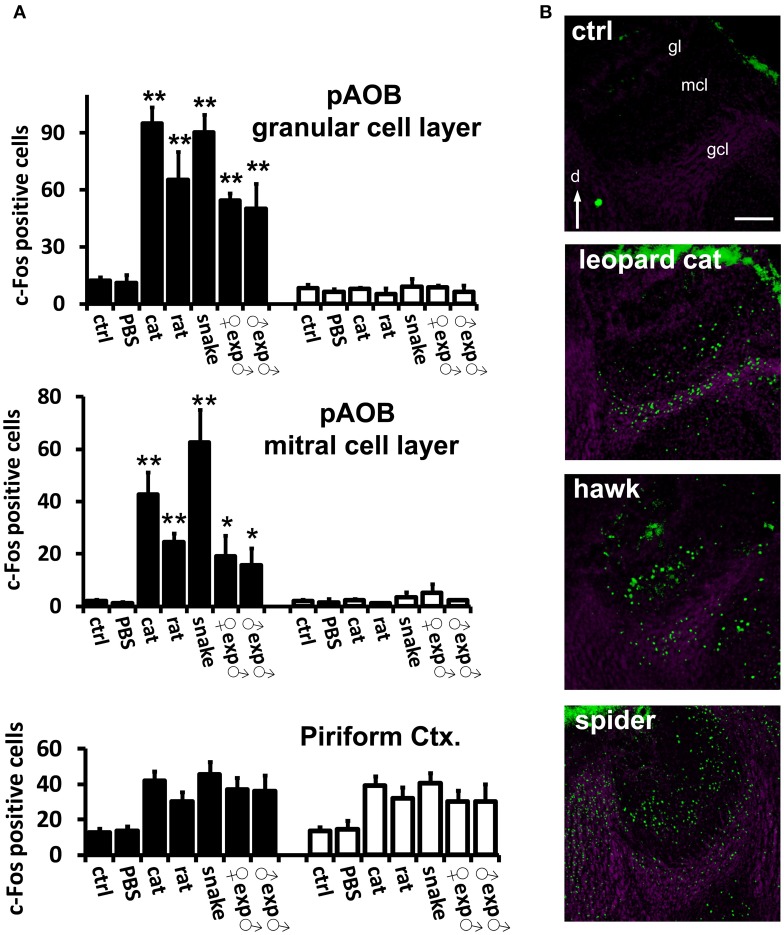
**Confirmation of VNO-mediated stimulus detection by investigation of activity in the accessory olfactory bulb in the brain. (A)** Quantification of odor-evoked c-Fos expression in the AOB in TrpC2^+/+^ (black bars) or TrpC2^−/−^ (white bars) genotypes. pAOB means activity in the posterior AOB. The top bar graph means activity in the granule cell layer and the middle graph represents activity in the mitral cell layer. Control odor is PBS-soaked or clean gauze for liquid or solid stimuli, respectively. *n* = 8–20. ^*^p < 0.05; ^**^p < 0.01. Mean ± s.e.m. ANOVA followed by Tukey-Kramer HSD *post-hoc* analysis (comparison of TrpC2^+/+^ against TrpC2^−/−^ for each odor). See Table S2 for numbers of c-Fos expressing cells and for other odors, and for a full description of controls, in both genotypes. Activation by each test stimulus and loss of activity in the mutants confirm that the stimuli activate the VNO and connected accessory olfactory pathway in the brain. Activity in the piriform cortex, which is not known to receive massive inputs from the vomeronasal organ, is not affected by the genetic ablation of the VNO in TrpC2^−/−^ animals (bottom graph). **(B)** Examples of immunostaining for c-Fos (green fluorescence) showing activation of the posterior division of the AOB in animals exposed to three taxonomically distantly related species (heterospecific odors). Purple labeling shows nuclear staining. gcl, granule cell layer; mcl, mitral cell layer; gl, glomerular layer; d, dorsal. Scale bar represents 100 μm. See Table S1 for a description of stimuli, and Table S2 for quantification of AOB activation. See also Papes et al. (2010), for images of AOB c-Fos immunostaining for other stimuli.

Interestingly, we noticed that heterospecific signals tended to activate the AOB and MeA more strongly than conspecific odors (Figure 1D), and we found a statistically significant positive correlation between activity induced by each stimulus in these areas and the number of *Egr1*-expressing cells in the VNO (Figures 2A,B; see also Figures 2C,D for control brain areas).

### Investigation of activity in the AOB and MeA after exposure to intra- and interspecies pure stimuli

Next, we intended to confirm that the observed activity in the MeA was due to pheromones and kairomones, by using pure ligands instead of complex odorous mixtures (see Materials and Methods section for details on pure stimuli). Recombinant versions of Mup proteins (rMups) were employed, namely, mouse pheromone rMups, which are able to induce aggressive and territorial behaviors (Chamero et al., 2007; Kaur et al., 2014), and a predator kairomone rMup, which triggers defensive behaviors (Papes et al., 2010).

First, we confirmed that neural activity due to each pure rMup is indeed part of that induced by the corresponding native stimulus, because exposure to a combination of each rMup plus the respective complex odor resulted in c-Fos counts which are not the sum of c-Fos positive cells in animals separately exposed to the pure or to the native stimuli alone (Figure 3A). Importantly, we found that equal amounts of predator (cat or rat) or mouse rMups elicited similar activity in the AOB or MeA (Figures 3A,B). This first-hand comparison of brain activity due to different pure stimuli revealed that the MeA, the activity of which correlates with VNO activation induced by complex stimuli (Figures 1, 2), is indeed activated by pure pheromones and kairomones.

### The medial nucleus of the amygdala receives major functional inputs from the VNO

In order to determine if the observed MeA activity was dependent on the VNO-mediated detection of pheromones/kairomones, we adopted a genetic strategy, where MeA c-Fos analysis was performed in TrpC2^−/−^ mutant animals without a functional VNO (see Materials and Methods for details on the knockout line).

**Figure 5:**
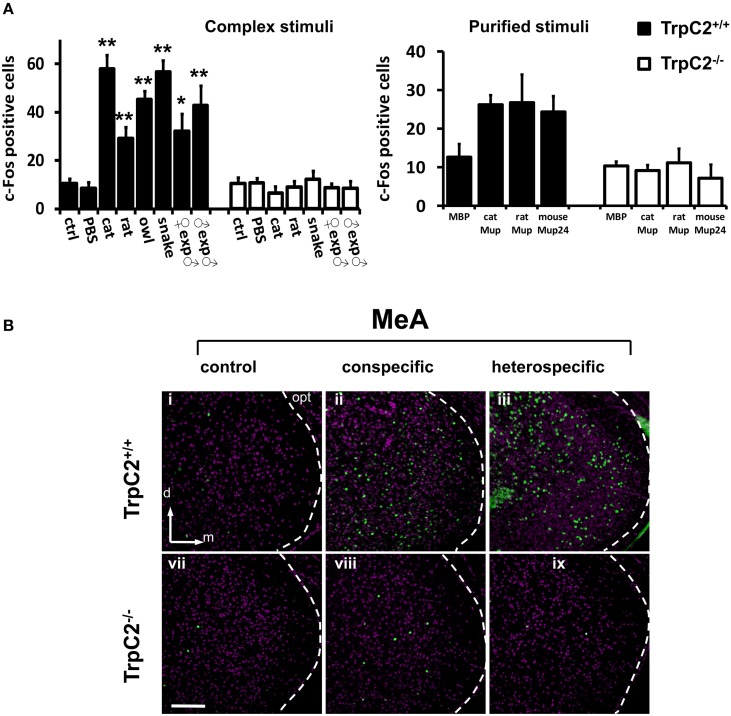
**Investigation of activity in the medial nucleus of the amygdala after exposure to odors detected by the VNO. (A)** Quantification of odor-evoked c-Fos expression in the MeA in TrpC2^+/+^ (black bars) or TrpC2^−/−^ (white bars) genotypes. The left bar graph shows data for exposures to native (complex) conspecific or heterospecific stimuli; the right bar graph exhibits data for purified stimuli. Control odor is PBS-soaked or clean gauze for liquid or solid stimuli, respectively. *n* = 8–20. ^*^p < 0.05; ^**^p < 0.01. Mean ± s.e.m. ANOVA followed by Tukey-Kramer HSD *post-hoc* analysis (comparison of TrpC2^+/+^ against TrpC2^−/−^ for each odor). See Table S2 for numbers of c-Fos expressing cells and for other odors, and for a full description of controls, in both genotypes. **(B)** Exposure to conspecific and heterospecific stimuli leads to activation in the MeA, judged by c-Fos expression (green). This effect is significantly impaired in TrpC2^−/−^ animals, where the VNO is non-functional. The white dashed lines mark the boundaries for the MeA. d, dorsal; m, medial; opt, optic tract. Scale bar represents 100 μm.

First, we confirmed that instinctive behaviors toward a large variety of hetero- or conspecific odors were impaired in TrpC2^−/−^ mutants (data not shown; see also Stowers et al., 2002; Chamero et al., 2007; Papes et al., 2010). Next, we found that the behavioral defects in the mutant animals were accompanied by severely reduced or abolished c-Fos expression in the AOB (Figure 4A and Table S2; see also Figure 4B and (Papes et al., 2010) for representative images of the pAOB activation after exposure to various odors), consistent with the notion that this brain region mainly collects information from the VNO (Wagner et al., 2006). Importantly, in the MeA, the number of c-Fos positive cells is much lower in TrpC2^−/−^ than in TrpC2^+/+^ mice, being comparable to unscented controls (Figure 5A, left graph for complex native stimuli and right graph for pure stimuli, and Figure 5B for representative images from animals of both genotypes exposed to conspecific or heterospecific odors; see also Table S2), suggesting that the observed increase in c-Fos expression in wild-type animals primarily results from processing of VNO signals.

It is important to note that other regions, such as the piriform cortex, another olfactory area in the brain, do not exhibit changes in activity in TrpC2^−/−^ animals (Figure 4A, bottom graph), in keeping with the notion that this brain region does not receive major inputs from the VNO (Stettler and Axel, 2009). It further shows that loss of activity is not widely distributed in the brains of TrpC2^−/−^ mice, strengthening the idea that the decrease in MeA activity in the mutants is specific and due to the lack of a functional VNO.

### Olfactory stimuli activate broadly distributed ensembles of neurons in the medial amygdala

**Figure 6:**
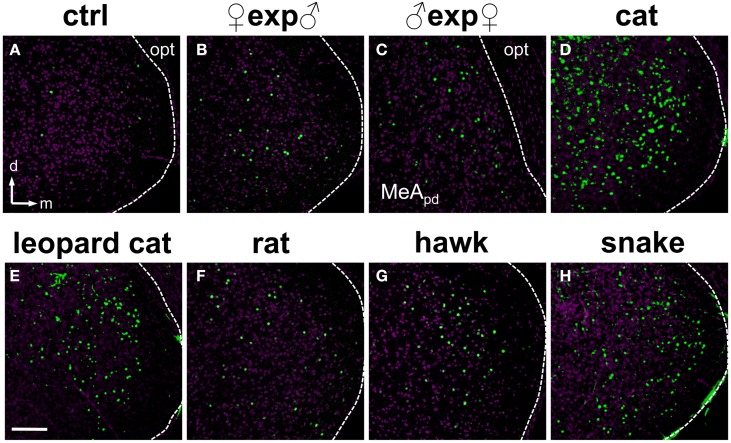
**Olfactory stimuli activate dispersed ensembles of cells in the medial nucleus of the amygdala. (A)** MeA activity observed in animals exposed to control odor (ctrl). **(B–H)** Distributed patterns of c-Fos expression in the posterior aspect of the MeA in animals exposed to conspecific **(B,C)** or heterospecific **(D–H)** stimuli, including several predator species. Panel **(C)** represents the dorsal portion of the MeA (MeApd), near the optic tract. Remaining panels represent the whole MeA. The white dashed lines mark the boundaries for the MeA. *n* = 4–6. d, dorsal; m, medial; opt, optic tract. Scale bar represents 100 μm.

**Figure 7:**
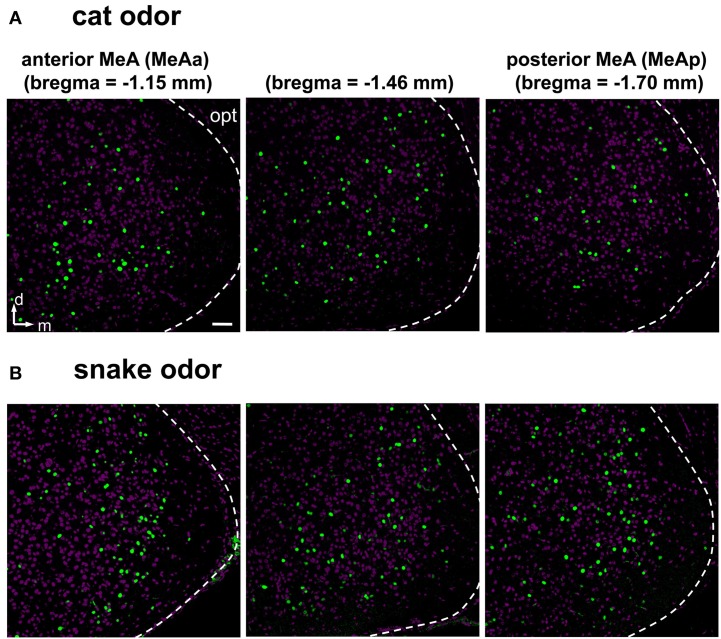
**Examples of activity in the medial nucleus of the amygdala along the rostral-caudal axis after exposure to heterospecific stimuli. (A,B)** Activation in the MeA is distributed across the nucleus and along the rostral-caudal axis (sections in three bregma positions are shown), in animals exposed to cat **(A)** or snake **(B)** odors. Images represent immunostaining for the marker of neuronal activation c-Fos (green fluorescence). Purple labeling, nuclear stain. The white dashed lines mark the boundaries for the MeA. d, dorsal; m, medial; opt, optic tract. Scale bar represents 100 μm.

**Figure 8:**
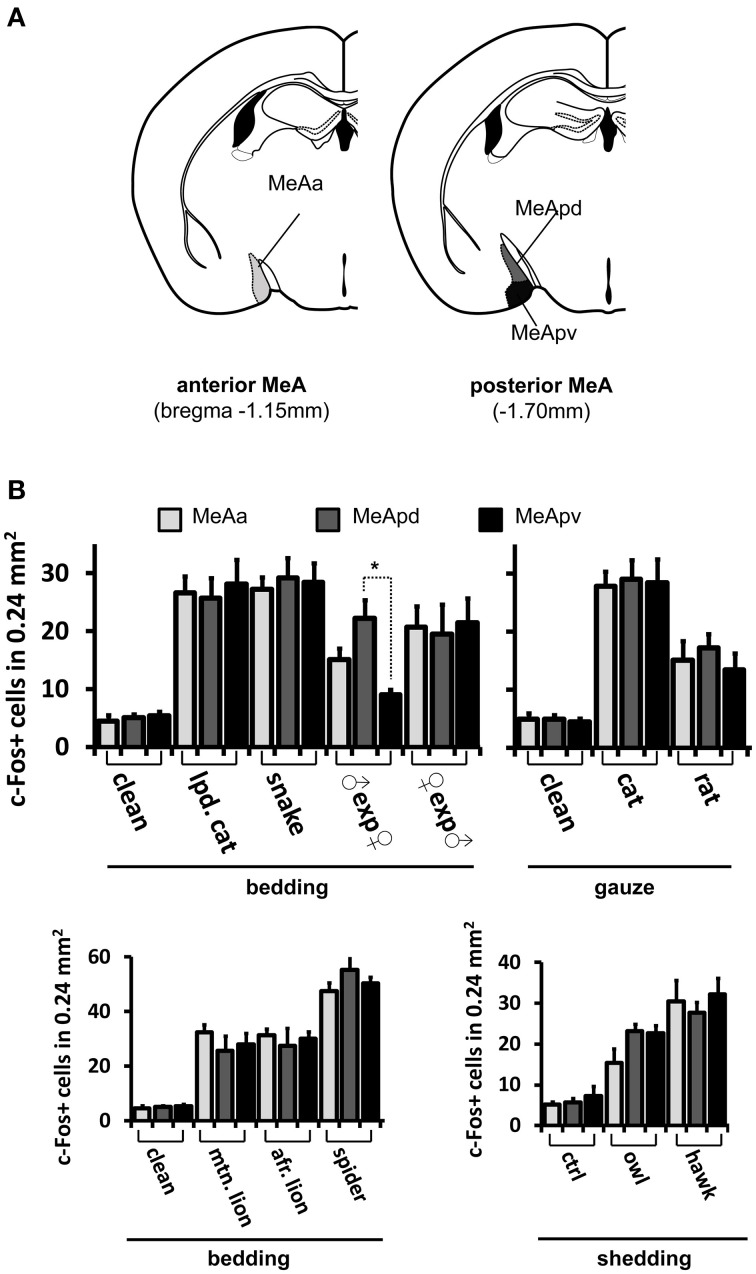
**Activity in the medial nucleus of the amygdala after stimulation with pheromones or kairomones is not spatially segregated. (A)** Schematic representation of the anatomical divisions of the MeA, showing its anterior (left) and posterior (right) aspects and the different subsectors therein, such as the anterior MeA (MeAa), posterodorsal MeA (MeApd), and posteroventral MeA (MeApv). **(B)** Distribution of activity in MeAa, MeApd, and MeApv parts of the MeA, for several stimuli (grouped by presentation form). ^*^p < 0.01. Mean ± s.e.m. ANOVA followed by Tukey-Kramer HSD *post-hoc* analysis. See also Figures 6, 8 for representative c-Fos images and other stimuli. lpd. cat, leopard cat; mtn. lion, mountain lion; afr. lion, African lion. ♀exp♂ denotes a female mouse exposed to male odors. In most cases, activity is dispersed in the MeA, without spatial segregation in the three subsectors, with the exception of a conspecific stimulus, namely, in males exposed to female odors.

Next, we investigated and compared how distinct stimuli lead to activation patterns in the MeA, by carefully evaluating the spatial distribution of activated cells in animals exposed to our wide set of heterospecific and conspecific stimuli. Overall, an average of 10–25% of MeA cells were activated, depending on the stimulus. In mice exposed to each of the predator odors, c-Fos-expressing cells were found broadly distributed in the MeA (Figures 6D–H). For such heterospecific stimuli, the distributive pattern was seen in both the dorsal and ventral aspects of the posterior MeA (MeAp) and in the anterior MeA (MeAa) (examples in Figure 6; see also Figure 7 and quantitation in Figure 8).

Strikingly, for most conspecific signals, the same broad distribution pattern was observed (Figures 6B,C), in both the dorsal and ventral aspects of the MeAp and in the anterior MeA (MeAa) (Figures 7, 8). In male mice exposed to female odors, the activated cells were found in both the dorsal and ventral MeAp, but with a higher concentration in the dorsal aspect (Figures 6C, 7B).

### The active ensembles of neurons in the MeA are not invariant

**Figure 9:**
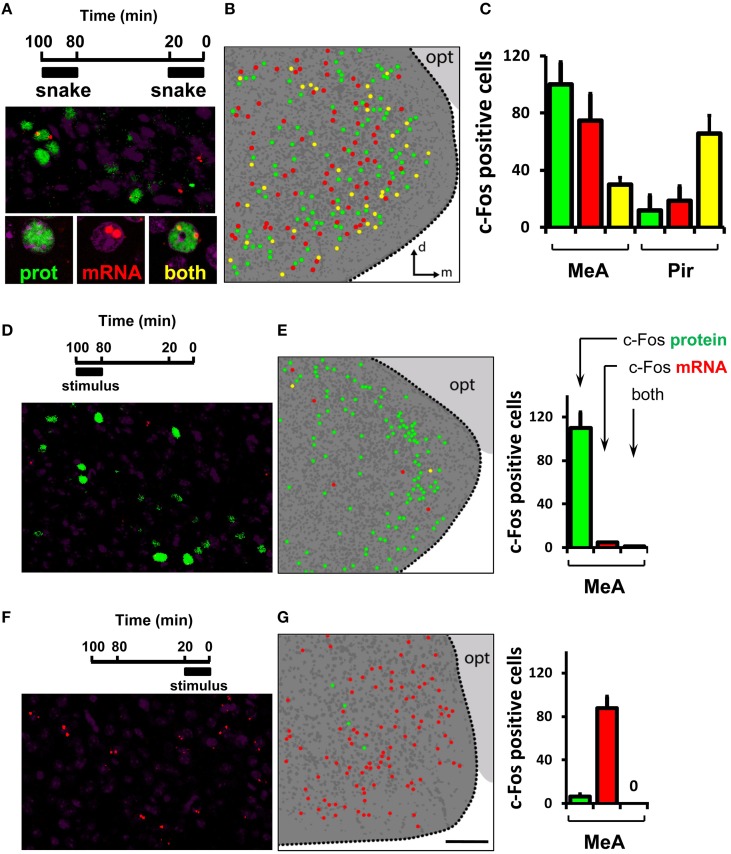
**Activity in the medial nucleus of the amygdala after detection of a VNO stimulus is not invariant. (A)** Dual immunostaining/*in situ* hybridization to compare activity related to two sequential exposures to the same olfactory stimulus. Top, exposure protocol. Bottom, representative image showing location of cells activated in the first exposure (green nuclear fluorescence after c-Fos immunostaining), in the second exposure (two red nuclear fluorescent foci after *c-Fos* FISH) or both (yellow). **(B)** Schematic drawing of a representative image of activated cells in the MeA after two sequential exposures to the same stimulus, collected across a 30 μm thick z-series. Dark gray dots represent nuclei of non-activated cells inside the MeA. Dark gray background indicates the medial amygdala as depicted in Figure 8A; light gray background marks the optic tract (opt). **(C)** Quantification of activated cells related to each of two instances of stimulus exposure. The first bar (green) in each set represents cells activated in the first exposure only, the second (red) bar in each set exhibits activated cells in the second exposure only, and the third bar (yellow) in each set represents cell activated in both exposures. Quantification of cells in the piriform cortex (Pir) is shown for comparison. **(D)** Dual immunostaining/*in situ* hybridization to control for activity related to one exposure to olfactory stimulus during the first application window (100 to 80 min prior to brain fixation). Top, exposure protocol. Bottom, representative image showing location of cells activated in the first exposure (green nuclear fluorescence after c-Fos immunostaining). Rare nuclear red fluorescence foci indicate *c-Fos* mRNA detected by FISH. Yellow cells express both nuclear c-Fos protein and mRNA. **(E)** Left, schematic representation of activated cells in the MeA after one exposure to stimulus during the first application window, collected across a 30 μm thick z-series. Right, quantification of activated cells related to stimulus exposure. The first bar (green) in each set represents cells activated in the first exposure only, the second bar (red) in each set exhibits rare cells expressing *c-Fos* mRNA, and the third bar (yellow) represents very few cells expressing both nuclear c-Fos protein and mRNA, indicating that *c-Fos* mRNA labels cells activated during the second exposure, because virtually no mRNA derived from the first exposure remains at the end of the session. (**F,G**) Same as in (**D,E**), but to examine activated cells related to one exposure to stimulus during the second application window (20 to 0 min before brain fixation), indicating that c-Fos protein is indicative of the first exposure window, because not enough time transpired after the second exposure onset to allow c-Fos protein to be synthesized at any detectable level. *n* = 4–6. d, dorsal; m, medial; opt, optic tract. Scale bar represents 100 μm; for (**B**, **E**), the scale is the same as in **(G)**.

Next, we investigated the stereotypy and invariance of the ensemble of activated cells in the MeA. To this end, we developed a dual c-Fos immunostaining/*in situ* hybridization protocol to parse out, in the same animal, the sets of active neurons related to two sequential olfactory exposure events (see Materials and Methods for details). In these experiments, c-Fos protein is indicative of activation during the first exposure, while *c-Fos* mRNA labels cells activated after the second exposure (Figures 9D–G).

Exposure of mice to the same stimulus twice leads to largely distinct subsets of activated cells in the MeA, with 20–30% overlap (Figures 9A–C; see also Video S1 for an explanation of dual staining visualization). This contrasts with the level of overlap we observed between groups of activated cells in the piriform cortex related to two instances of exposure to the same stimulus: in this area, the same stimulus resulted in the consistent activation of largely overlapping groups of neurons over two subsequent trials (Figure 9C, right bars, Pir): 83.7 ± 1.7% of all piriform cells expressing c-Fos protein also express *c-Fos* mRNA; and 77.2 ± 2.1% of all cells expressing *c-Fos* mRNA also express c-Fos protein.

Interestingly, the concordance level between the active MeA ensembles in two exposures to the same stimulus is significantly larger than that expected by chance alone [23.5 ± 0.8% of all cells expressing c-Fos protein that also express *c-Fos* mRNA vs. 1.1 ± 0.2% overlap expected by chance (chi-square test of goodness-of-fit; *P* = 0.85); or 27.8 ± 1.9% of all cells expressing *c-Fos* mRNA that also express c-Fos protein vs. 0.96 ± 0.3% overlap expected by chance (*P* = 0.82)]. Together, the foregoing results suggest that the active ensembles in the MeA, though not random, are not invariant.

### Distinct olfactory stimuli activate intermingled ensembles of neurons in the medial amygdala

**Figure 10:**
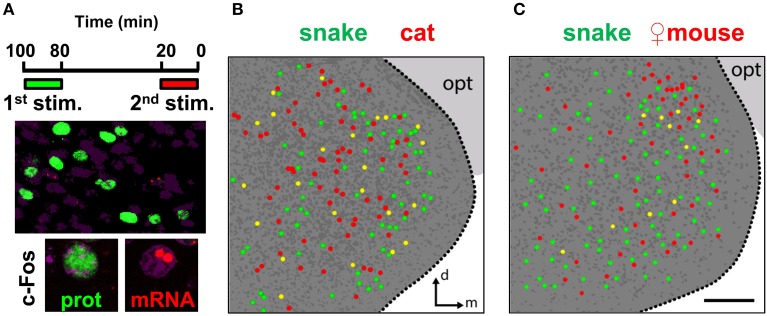
**Distinct stimuli activate intermingled ensembles of neurons in the medial nucleus of the amygdala. (A)** Dual immunostaining/*in situ* hybridization to compare activity in the MeA related to two sequential exposures to distinct olfactory stimuli. Top, exposure protocol. Bottom, representative images showing cells activated in the first exposure (green nuclear fluorescence after c-Fos immunostaining) and in the second exposure (two red nuclear fluorescent foci after c-Fos FISH). **(B)** Schematic drawing of a representative image of activated cells in the MeA after two sequential exposures to distinct predatory olfactory stimuli, collected across a 30 μm thick z-series. Dark gray dots represent nuclei of non-activated cells inside the MeA. Dark gray background indicates the medial amygdala; light gray background marks the optic tract (opt). **(C)** Same as in **(B)**, but to examine activated cells related to sequential exposures to a predator odor and conspecific odor. *n* = 4–6. d, dorsal; m, medial; opt, optic tract. Scale bar represents 100 μm; for **(B)**, the scale is the same as in **(C)**.

To directly compare the spatial organization of activity in the MeA due to distinct stimuli (Figure 10), we used the dual c-Fos immunostaining/*in situ* hybridization protocol described above. When we compared snake and cat stimuli, the overlap seen between the active MeA ensembles during the two exposures was not small [25.2 ± 2.4% of all cells expressing c-Fos protein that also express *c-Fos* mRNA vs. 1.5 ± 0.4% overlap expected by chance (chi-square test of goodness-of-fit; *P* = 0.81); or 23.8 ± 0.9% of all cells expressing *c-Fos* mRNA that also express c-Fos protein vs. 1.4 ± 0.4% overlap expected by chance (*P* = 0.90)]. Similar results were obtained in the comparison between snake (heterospecific) and female mouse (conspecific) odors (not shown). Although significantly larger than that expected by chance alone, this overlap level does not permit us to easily and unequivocally interpret whether different stimuli activate non-overlapping ensembles or whether the active sets of cells have some degree of overlap in the MeA, because the same stimulus induces c-Fos expression in subsets of cells with an equivalent degree of overlap over two subsequent trials (Figure 9C).

However, the comparison between multiple stimuli with our dual staining protocol does enable us to determine if there is spatial segregation of activated cells in the MeA. We did not verify any immediately discernible differential distribution of activated cells when one predator stimulus was compared to a different predator stimulus (Figure 10B), indicating that the active ensembles related to exposures with these distinct stimuli were intermingled in broad regions of the MeA. Similarly, we could not verify spatial segregation in the MeA when a predator odor was compared in the same animal with odors from conspecific same-sex individuals: in males, the comparison between a predator stimulus and female mouse odors (which activate cells distributed in the entire MeA but concentrated along its dorsal side) also revealed that these two stimuli activate mostly interspersed groups of cells throughout the MeA (Figure 10C).

### Organization of activity in the MeA does not reflect behavioral output, stimulus valence, stimulus origin, chemical category or receptors activated at the sensory organ level

**Figure 11:**
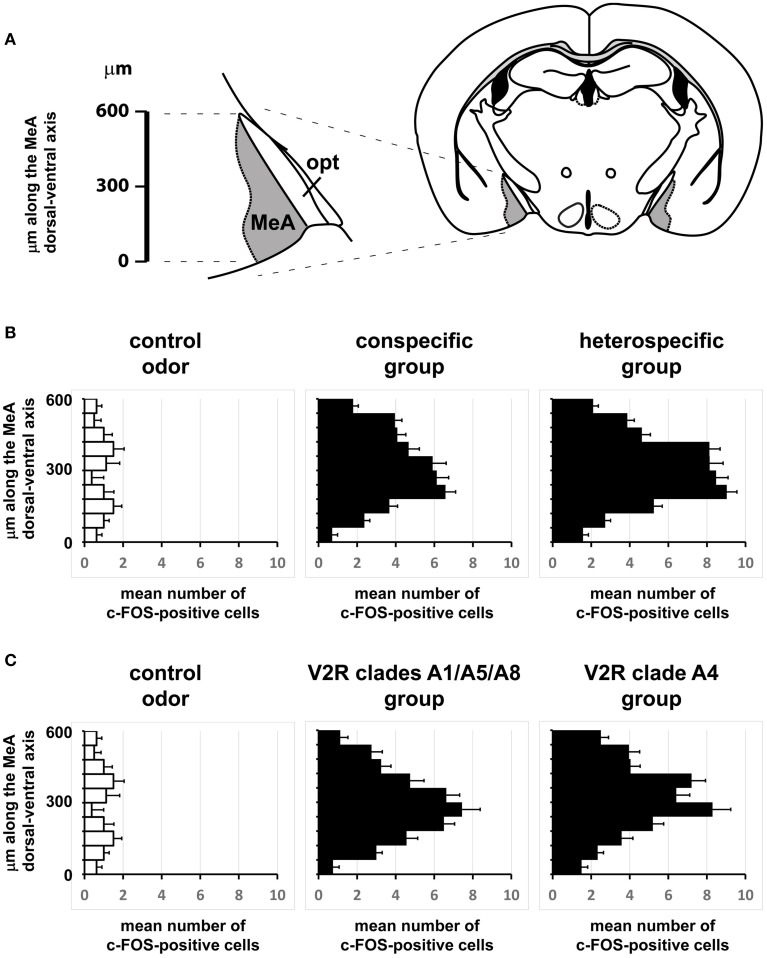
**Comparisons of the spatial distributions of activated cells along the dorsal-ventral axis of the medial nucleus of the amygdala. (A)** Schematic diagram showing how the dorsal-ventral axis was positioned in the MeA. The axis origin (0) was defined as the most ventral pixel in the MeA in each scored image, while the upper extreme at the dorsal side lies 600 μm from the origin along the dorsal-ventral axis. This segment was further subdivided into 10 intervals and activated cells were counted for each interval in each image. **(B)** Horizontal bars represent the mean number of activated cells in each of the 10 intervals along the MeA axis (600 μm in total length), leading to a representation of the spatial distribution of the active ensemble. Black bars in the middle graph were obtained with data from mice exposed to conspecific stimuli (♂exp♀, ♀exp♂, ♀exp♀, and ♂exp♂ groups); black bars in the right graph are for mice exposed to heterospecific odors (cat, leopard cat, mountain lion, rat, owl, hawk, and snake); white bars in the left graph represent basal level activation with control odors (see Table S2). *n* = 8 for each species. Error bars are s.e.m. **(C)** Similar to **(B)**, but comparing MeA activation after exposure to odors that activate VNO neurons expressing A1/A5/A8 V2R receptors (♂exp♀, ♀exp♂, and ♂exp♂ groups) against the group of animals exposed to stimuli related to A4 V2R receptors (cat, leopard cat, and mountain lion). *n* = 8 for each species. Error bars are s.e.m.

If distinct olfactory stimuli activate intermingled ensembles of cells in the MeA, without spatial order, how is activity organized in this nucleus? Odors capable of eliciting defensive responses activated both the dorsal and ventral MeA (Figures 6–8), and therefore activity related to defensive stimuli is not exclusively restricted to or markedly enriched in the ventral MeA, as previously suggested (Swanson, 2000; Canteras, 2002; Choi et al., 2005). The spatial distributions of cells in the active ensembles are not significantly different for heterospecific vs. conspecific stimuli (Figure 11B; two sample Kolmogorov-Smirnov distribution comparison (KS) test, *P* = 0.26; see Materials and Methods and Figure 11A for details). Therefore, the organization of MeA activity does not seem to reflect the source of stimulus origin (heterospecific vs. conspecific) or the behavioral outputs (reproductive vs. defensive).

Second, we alternatively hypothesized that activity in the MeA could be organized according to the taxonomic groups to which the species tested belong. Even though the numbers of distinct species used in each taxonomic group were limited, our data do not support this model, because the distributions of activated cells for avian predators (Figures 6G, 8C), mammals (Figures 6D–F, 7B, 8A,C), reptiles (Figures 6H, 7B, 8B), and even invertebrates (Figure 8B) are all dispersed across the nucleus, without preference for dorsal or ventral MeA, or for pMeA or aMeA (Figure 8).

Third, we examined whether activity in the MeA is dependent on the chemical nature of the stimuli. For example, small organic molecules could be represented in one MeA sector, while proteins/peptides would evoke activity in another. However, our data show that male mouse urine (containing both Mup proteins and small organic VNO ligands; Chamero et al., 2007) activates a distributed ensemble (Figures 5B, 6B,C, 8B), whereas cat odor (containing a defensive behavior-inducing Mup; Papes et al., 2010) produces a similar pattern (Figures 5B, 6D, 8B). Therefore, our data indicate that stimuli of dissimilar chemical nature do not necessarily induce distinct patterns of MeA activity, suggesting that the mapping of olfactory information in this nucleus is not dependent on the chemical class to which the stimulus belongs.

Finally, we verified whether activity in the MeA is dependent on the molecular identity of activated sensory VNO neurons. Previous publications (Isogai et al., 2011) indicate that most stimuli employed in our study are detected by cells in the basal zone of the VNO, characterized by the expression of GPCR receptors in the V2R family (~120 family members, phylogenetically grouped into several clades; Silvotti et al., 2007). Therefore, we decided to focus on defining the V2R receptors expressed in activated VNO neurons in animals individually exposed to each stimulus, using double fluorescent *in situ* hybridization with one probe to detect the expression of the marker of vomeronasal neuron activation *Egr1* and other probes to detect specific clades of V2R receptors (Figure S1A) (see Materials and Methods for probe design and validation).

Stimuli that activate VNO neurons expressing V2R receptors in the A4 clade (nomenclature following Silvotti et al., 2007), such as feline odors, all produce distributed c-Fos expression in the MeA (Figures 6D,E, Figures S1B, S1C, right panel). Chemosignals detected by VNO cells expressing clades A8, A5, and A1 of V2R receptors, such as same or opposite-sex adult mouse odors (Figures 6B,C and Figure S1B), also activate dispersed ensembles in the MeA. The ensembles activated by odors belonging to these two categories are in fact intermingled (Figures 9, 10), and the distributions of activated cells (Figure 11C) are not statistically significantly different between the two groups (two-sample Kolmogorov-Smirnov test; *P* = 0.40).

## Discussion

### Representation of olfactory information in the brain

In this study, we investigated the activity in the mouse medial amygdala induced by odors that elicit instinctive behaviors important for the survival of the individual and the species (pheromones and kairomones). Using a combination of genetic and brain activity analyses, we show that a wide range of different odors activate cells in the MeA, and that this area is functionally involved in the circuit initiated at the VNO. Though the general involvement of the amygdala in processing olfactory information has been appreciated for some time (Swanson, 2000; Takahashi, 2014), its exact place in the neural mapping and representation of odors remains poorly characterized. We investigated the organization of activity in this area and found that there is a lack of spatial segregation for activity related to distinct stimuli, without reflecting output behaviors or stimulus valence, which is in opposition to the current views about how activity in the medial amygdala is laid out.

### Activation of the medial amygdala by a large repertoire of intra- and interspecific odors

In this study, we comparatively analyzed brain activity in the MeA by using a wide range of intra- and interspecies olfactory stimuli (Table S1) which are able to induce instinctive behavioral responses in mouse subjects, including aggression, sexual behavior, or defensive behavior (Figure 1A). Curiously, we found that several heterospecific odors trigger defensive behaviors, greatly expanding the previously described list of kairomones (Papes et al., 2010) and evidencing the importance of olfaction in the detection of danger. In animals exposed to most conspecific and heterospecific stimuli, a significant subgroup of VNO sensory neurons was activated. We noticed that heterospecific odors that induce defensive behaviors tended to activate more VNO cells than other types of stimuli (Figure 1B), a result that stresses the importance of the mammalian VNO in the detection of odors from other species (Papes et al., 2010; Isogai et al., 2011).

An investigation of activity in the AOB and MeA in mice exposed to such chemosignals revealed that these two areas are activated by those VNO stimuli (Figures 1, 2). Direct projections of VNO neurons to the AOB are known since the times of Ramon y Cajal, and indirect anatomical links between the MeA and VNO have been known for a while (Canteras et al., 1995, 1997; Petrovich et al., 2001; Blanchard et al., 2005; Mohedano-Moriano et al., 2007). Our data now provide a comprehensive view of how the AOB and MeA are activated by odorous stimuli, revealing that a strikingly large set of behavior-inducing odors activates these areas. Additionally, we found that these nuclei are more strongly activated by heterospecific odors than by conspecific stimuli (Figure 1D), revealing the unprecedented fact that the mammalian brain is exquisitely activated by odors from other species in the environment. Finally, we show that activity in the MeA is correlated with the detection of stimuli by the VNO (Figure 2), supporting the idea that it belongs to a functional pathway initiated at the VNO to process olfactory information.

Because most stimuli employed in these experiments were complex in nature, we needed to confirm that pheromones and kairomones contained therein could indeed activate the circuit initiated at the VNO, including the AOB and MeA. In fact, when we used recombinant versions of Mup proteins from mouse and cat, which act as pheromones and kairomones, respectively (Chamero et al., 2007; Papes et al., 2010), the VNO, AOB, and MeA were activated (Figure 3).

By using a wide variety of behavior-inducing odors, together these experiments (Figures 1–3) led to the corroboration that the MeA is activated by pheromones and kairomones, and greatly expanded the known repertoire of signals, both complex and pure, able to provide sensory input to this region; moreover, these data suggest that the MeA mostly receives functional inputs from the VNO, because MeA activity is highly correlated with VNO detection of behavior-inducing odors.

### Functional links between MeA and VNO

The activity we observed in the MeA with complex olfactory stimuli (Figure 1) may not necessarily be dictated by the VNO, because MeA neurons may also receive inputs from other sensory organs. In this vein, we noted that the piriform cortex, which does not receive major inputs from the VNO, is also activated by our complex stimuli (Figure 1D). Therefore, we needed to establish the existence of possible functional links between the observed MeA activity and VNO detection of pheromones/kairomones. In order to do this, we exposed VNO-deficient TrpC2^−/−^ mice to the chemosignals described in the previous section. We observed that the expression of the indirect marker of MeA neuronal activation c-Fos was much lower in TrpC2^−/−^ than in TrpC2^+/+^ mice, similar to the expression seen in animals exposed to unscented controls (Figure 5), indicating that MeA activation is functionally linked to the detection of stimuli at the VNO sensory interface.

In combination with the fact that activity in the MeA is highly correlated with VNO activity (Figures 1, 2), these results in TrpC2^−/−^ animals indicate that the MeA is mostly controlled by the vomeronasal system, favoring the model in which it is part of a functional circuit initiated at the VNO to control behavior (Canteras et al., 1997; Swanson, 2000; Blanchard et al., 2005; Takahashi, 2014).

### Organization of odor-induced activity in the medial amygdala

In our study, the use of a wide selection of heterospecific and conspecific signals and of potent stimuli from several species enabled us to conclude that each tested stimulus induces c-Fos expression in cells broadly distributed throughout the MeA, with no apparent spatial segregation (Figures 6–8). Such data suggest that different stimuli do not result in grossly spatially segregated ensembles of active neurons in this nucleus of the amygdala. We also investigated activity in the granule and mitral cell layers of the AOB, and, similarly to the MeA, we found it not to be organized in a spatially segregated fashion (defensive and social stimuli produced similarly distributed activity), in keeping with previous publications (Luo et al., 2003; Hendrickson et al., 2008; Ben-Shaul et al., 2010). These data suggest that the distributive activity in the AOB is maintained in the MeA, the first information processing station after the olfactory bulb in the brain.

We further showed that different VNO stimuli activate intermingled ensembles of cells in the MeA, without evident spatial separation (Figures 6, 10). The apparent lack of segregation of activated ensembles for different olfactory stimuli indicates that a spatial map to internally represent VNO ligands is not formed in the MeA (or that its underlying organization is undiscernible at this resolution).

These results put into question previous ideas according to which the MeA would be functionally divided into two sectors, dorsal and ventral, and stimuli related to distinct behavioral outputs (namely, reproductive or defensive) would activate spatially segregated groups of neurons in those two sectors, respectively (Swanson, 2000; Canteras, 2002; Choi et al., 2005). Although previous anatomical and functional evidences suggested that some stimuli may induce coherent activity in the MeA consistent with this notion (Choi et al., 2005), the use of a wide range of stimuli in our study enabled us to rule out this model by showing that distinct stimuli activate an equivalent percentage of MeA neurons and that they are mostly intermingled, without apparent spatial segregation.

Though stimuli that induce distinct behavioral outputs (defensive vs. reproductive) were not found to be spatially segregated in a consistent fashion, it remains possible that these two sets of cells send information to different downstream areas, resulting in distinct coding and processing pathways to trigger opposing behaviors. In fact, the active MeA ensembles related to cat and female odors were found to be projection neurons with largely distinct projection sites in the brain (Choi et al., 2005); moreover, tracing studies indicated, at a grosser level, that amygdala projection neurons target several brain areas, with some degree of overlap (Canteras et al., 1995; Dong et al., 2001; Petrovich et al., 2001; Canteras, 2002).

The distributive organization we observed in the MeA is also noteworthy in view of the fact that this nucleus is heterogeneous and contains cells selectively activated by particular ligand types: for example, some cells are active in animals exposed to predator odors, others are active in males exposed to females, others are activated by same-sex odors, while some are responsive to all kinds of stimulation (Bergan et al., 2014). It is therefore possible that the distinct subtypes of MeA neurons are intermingled, but projecting to distinct target output regions.

Interestingly, the distributive organization of MeA activity resembles the piriform cortex, where volatile odorants detected by the main olfactory epithelium are internally represented by distributed ensembles of active neurons, without any discernible spatial organization, irrespective of the stimulus' chemical nature, concentration or valence (Stettler and Axel, 2009). In both the piriform and MeA, each stimulus activates a percentage of the neurons, and the activated neurons are distributed, with no apparent spatial segregation or stereotypy. Moreover, different odors activate distinct, but partially overlapping, ensembles of neurons. The similarity between the representation of odors in the piriform and in the MeA concurs with the suggested cortical-like nature of some amygdalar areas.

Curiously, the cortical amygdala, which receives olfactory information collected by the main olfactory epithelium, seems to be organized in a spatially segregated fashion (Miyamichi et al., 2011; Sosulski et al., 2011). Moreover, volatile odorants that elicit innate behaviors of different valence (appetitive or aversive) activate different populations of neurons within the cortical amygdala (Root et al., 2014), suggesting that the representation in such brain region is different from the representation of pheromones/kairomones in the MeA.

We have also investigated the stereotypy of the active cells in the MeA, and found that neurons activated by exposure to one stimulus are mostly distinct from those activated by a later exposure to the same stimulus (Figure 9). These data show little concordance for the sets of MeA cells activated in response to the same stimulus in two trials, suggesting that determined or immutable ensembles of cells related to each VNO stimulus do not exist in this brain area; however, because the concordance is higher than that expected by chance alone, the active ensemble to one stimulus is probably not random.

The little concordance between the two groups of neurons activated by the same odor in the MeA is not due to restrictions imposed by the use of c-Fos as a marker for neuronal activation in our dual immunostaining/*in situ* hybridization technique, because a high level of overlap was seen for two sequential exposures to the same stimulus in another brain area, the piriform cortex, using the same procedure (Figure 9). Our data on the piriform cortex is in agreement with a previous publication, which verified that piriform neurons that respond to an odorant have a high chance of responding at least once again to the same odorant over subsequent trials (Stettler and Axel, 2009). It is interesting to note, however, that we observed higher levels of concordance in the piriform cortex than another study (Shakhawat et al., 2014), which used a similar dual staining procedure to evaluate responses in the primary olfactory cortex, but found that the same odor activates ensembles with < 30% overlap when given repeatedly; a possible reason for such difference is the fact that Shakhawat and collaborators employed purified odorants while we used complex stimuli, which activate a larger ensemble of piriform neurons, increasing the chances of overlap in a subsequent exposure trial. Further studies will be necessary to explore the difference between stereotypy in the MeA and piriform. If confirmed by additional methods (e.g., electrophysiology or functional imaging), the representation of odorants in the piriform and of pheromones/kairomones in the MeA will be different in one significant aspect: in the piriform, each odor stimulates activity in a distributed and sparse ensemble of neurons, but according to our data that ensemble is mostly defined (Figure 9); in contrast, our results show that the active ensembles in the MeA in response to conspecific/heterospecific stimuli are non-immutable (Figure 9).

Lastly, we show that the distributive pattern of activity in the MeA is not organized to reflect the type of stimulus employed, because no discernible differential distribution of activated cells is observed between animals exposed to heterospecific or to conspecific stimuli (Figure 10). Moreover, we show that MeA activity is not organized to reflect the different behavioral consequences of the detected stimuli (reproductive vs. defensive). These data are contrary to previous notions established for the organization of the medial amygdala (Swanson, 2000; Canteras, 2002; Choi et al., 2005) and call for alternative models to explain how pheromones and kairomones produce coherent activity in this brain region. We further showed that the MeA activity does not indicate the stimulus chemical nature or the repertoire of receptors expressed in activated neurons at the sensory interface (Figures 10, 11).

Further anatomical and functional studies will be needed to confirm that the MeA does not internally represent the ensuing behaviors after detection of chemosignals by the VNO. If confirmed, these findings will pose an exciting final question: where are the olfaction-mediated instinctive behaviors represented in the brain? The hypothalamus, particularly its ventromedial nucleus, the dorsal premammillary nucleus, and the periaqueductal gray, anatomically positioned downstream to the MeA (Motta et al., 2009), are likely candidates, since they are activated by pheromones/kairomones (Dielenberg et al., 2001; Meredith and Westberry, 2004; Choi et al., 2005; Lin et al., 2011) and have been causally implicated in numerous behaviors. In the future, functional mapping of the flow of olfactory information along this brain circuit will be needed to understand the transition between the dispersed amygdala activity we describe here and the representation and generation of adaptive behaviors in yet uncharacterized higher brain sites.

### Conflict of interest statement

The authors declare that the research was conducted in the absence of any commercial or financial relationships that could be construed as a potential conflict of interest.
