# Supplementary material for: Lack of spatial segregation in the representation of pheromones and kairomones in the mouse medial amygdala
Source: Front Neurosci. 2015 Aug 11;9:283. doi: 10.3389/fnins.2015.00283 (PMC4531233; doi:10.3389/fnins.2015.00283)
Supplement: Supplementary file 1 [file Table1.PDF]

Supplementary Material

Lack of spatial segregation in the representation of pheromones and kairomones in the mouse medial amygdala

Vinicius M. A. Carvalho, Thiago S. Nakahara, Leonardo M. Cardozo, Mateus A. A. Souza, Antonio P. C. B. R. Camargo, Guilherme Z. Trintinalia, Eliana Ferraz, and Fabio Papes*

*** Correspondence:** Corresponding author: Fabio Papes; papesf@unicamp.br

1. Supplementary Data

1.1. Supplementary Video 1. Schematic representation of dual c-Fos immunostaining / in situ hybridization method and results. The first segment shows the stimulation protocol and the image of a cell in the VMH where c-Fos protein is expressed in the nucleus (green), representing activation during the first olfactory stimulation period (with snake odors, in this case). The second segment shows the stimulation protocol and a z-series across the VMH depicting a cell where immature c-Fos mRNA is detected by fluorescent in situ hybridization in two nuclear foci, related to the second, most recent, exposure period (red). The z-series is followed by its 2D reconstruction. The third segment shows the stimulation protocol and a z-series across the VMH depicting a cell where both c-Fos protein and mRNA are detected by fluorescent in situ hybridization in the nucleus, related to the the first and second stimulations, respectively (green and red). The z-series is followed by its 2D reconstruction at the end. **Supplementary Video 1 is provided as a separate video file.**

2. Supplementary Figures and Tables

The Supplementary Material for this manuscript includes one Supplementary Figure (Figure S1; both the figure and the legend are found below) and two Supplementary Tables (Tables S1 and S2). Table S1 is provided in this word document, with its legend. Table S2, due to its large size, is provided as a separate worksheet file, but the legend is included in this Supplementary Material document.

2.1. Supplementary Figures

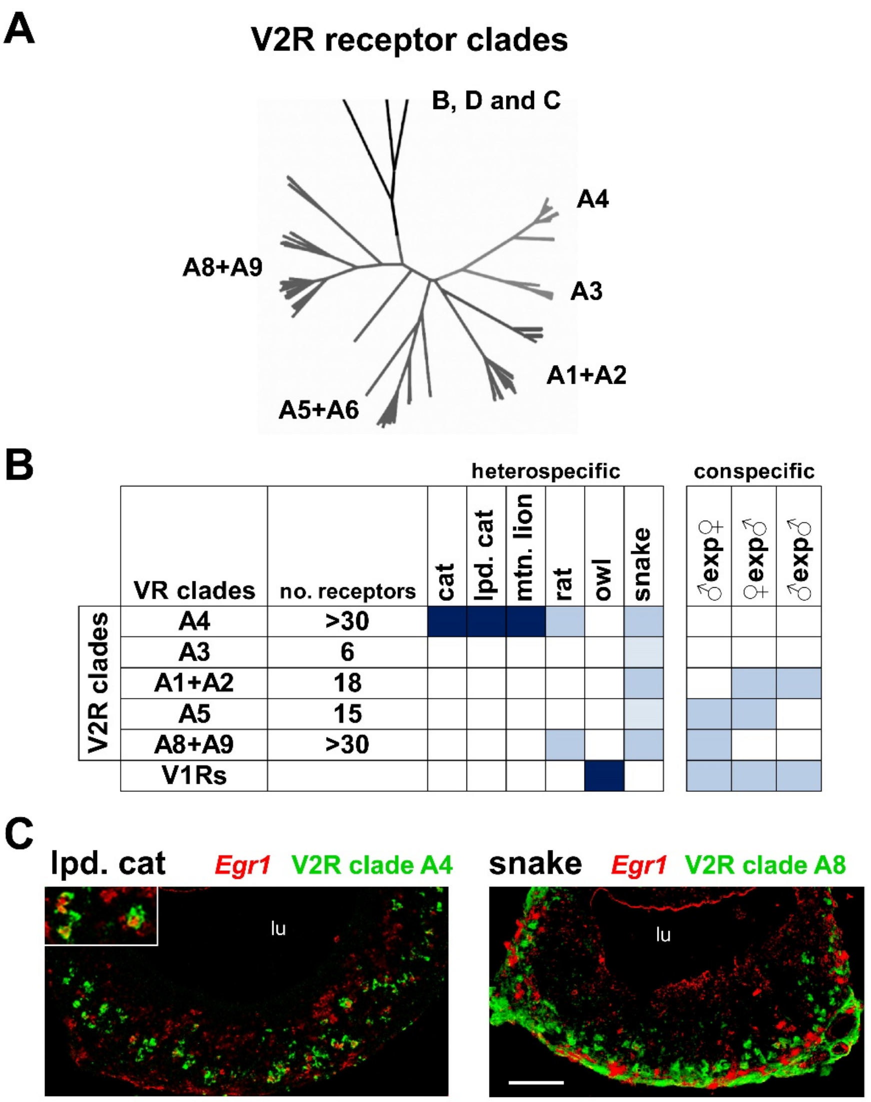

Supplementary Figure 1. Investigation of receptors in the V2R family expressed in vomeronasal sensory neurons activated by chemosignals. (A) Phylogenetic tree of V2R receptors, evidencing clustering into several clades. The figure focuses on A-type clades, following nomenclature used in Silvotti et al., 2007. (B) Heat map showing V2R receptors in several clades expressed in VNO neurons activated after exposure to various heterospecific and conspecific stimuli. The number of receptors in each clade is given in the second column. Dark blue indicates co-expression of Egr1 and the tested clade in more than 10 cells per VNO section. Intermediate blue hue indicates 3 to 10 co-labeled cells per section, lighter blue indicates 1 or 2 co-labeled cells per sections, and white indicates absence of co-labeling. Check also Isogai et al., 2011, for V2R receptors expressed in cells activated by other stimuli. (C) Examples of double *in situ* hybridization for *Egr1* (red) and vomeronasal receptors (green) in VNO sections of animals exposed to different stimuli, with labeling co-localization evidenced in inset. See Experimental Procedures for probe information and validation. lu, lumen. Scale bar represents 100 µm.

2.2. Supplementary Tables

2.2.1. Supplementary Table 1. List of olfactory stimuli used. Details for each heterospecific or conspecific stimulus used, including donating species information (common and scientific names), presentation forms (on gauze, as scented bedding or bodily sheddings), method of collection, amount used and type of behavior reported on mice.

	Common species name	Scientific name	Presentation form	Collection method	Amount used	Reported behavior on mice
**Crude stimuli**	Domestic cat	*Felis catus*	Gauze scented with bodily secretions	Gauze rubbed against the neck and mouth regions	one 8-ply 5 x 5 inches gauze	Fear behavior (Dielenberg et al., 2001; Papes et al., 2010)
	Domestic cat	*Felis catus*	Bodily shedding	Shaved cat fur	1 g	Fear behavior (Papes et al., 2010 and this study)
	Leopard cat	*Leopardus pardalis*	Scented bedding	Bedding collected directly from devoted cage	50 ml	Fear behavior (this study)
	Cougar mountain lion	*Puma concolor*	Scented bedding	Bedding collected directly from devoted cage	50 ml	Fear behavior (this study)
	African lion	*Panthera leo*	Scented bedding	Bedding collected directly from devoted cage	50 ml	Fear behavior (this study)
	Rat	*Rattus norvegicus* (Sprague-Dawley strain)	Scented bedding	Bedding collected directly from devoted cage	50 ml	Fear behavior (this study)
	Rat	*Rattus norvegicus* (Sprague-Dawley strain)	Gauze scented with bodily secretions	Gauze scented with liquid urine (1ml, unless otherwise noted)	one 8-ply 5 x 5 inches gauze	Fear behavior (Papes et al., 2010; this study)
	Great horned owl	*Bubo virginianus*	Bodily shedding	Feathers taken from cage	1 g	Fear behavior (this study)
	Crested caracara hawk	*Polyborus plancus*	Bodily shedding	Feathers taken from cage	1 g	Fear behavior (this study)
	Cornsnake	*Pantherophis guttatus*	Bodily shedding	Shed skin	1 g	Fear behavior (Papes et al., 2010; this study)

	Jararaca snake	*Bothrops jararaca*	Bodily shedding	Shed skin	1 g	Fear behavior (this study)
	Jararacussu snake	*Bothrops jararacussu*	Bodily shedding	Shed skin	1 g	Fear behavior (this study)
	Tarantula spider	*Theraphosidae* family	Scented bedding	Bedding from devoted cage	50 ml	Fear behavior (this study)
	Domestic rabbit	*Orictolagus cuniculus*	Gauze scented with bodily secretions	Gauze scented with liquid urine (1ml)	one 8-ply 5 x 5 inches gauze	No behavior on mice
	Mouse	*Mus musculus*	Scented bedding (male, unless otherwise noted)	Bedding collected directly from devoted cage	50 ml	
**Pure stimuli**	Cat	*Felis catus*	Purified stimulus (cat Mup = Feld4)	Gauze scented with purified stimulus	10 mg	Fear behavior (Papes et al., 2010; this study)
	Rat	*Rattus norvegicus*	Purified stimulus (rat Mup13)	Gauze scented with purified stimulus	10 mg	Fear behavior (Papes et al., 2010; this study)
	Mouse	*Mus musculus*	Purified stimulus (mouse Mup24)	Gauze scented with purified stimulus	10 mg	Aggression (Chamero et al., 2007)
	Mouse	*Mus musculus*	Purified stimulus (mouse Mups 24+8+3+25)	Gauze scented with purified stimulus	10 mg total (equal amounts)	

2.2.2. Supplementary Table 2. c-Fos cell counts in the brain of animals exposed to various stimuli. Each table cell represents the numbers of c-Fos-positive nuclei (mean ± s.e.m.) in TrpC2+/+ or TrpC2-/- littermates exposed to each stimulus indicated on the top row (‘odor’) or to respective control odors (‘ctrl’). The type of stimulus and respective olfactory controls are given in the last two rows for each stimulus. Abbreviations for each brain region follow those used in the Paxinos brain atlas (Paxinos and Franklin, 2004) and are the same used in the Figures and main text. On the rightmost part of each row, it is given the meanings of colors presented in heat maps in Figures 1 and 3 (numbers of c-Fos positive cells per unit area in each region). White color always corresponds to activity level observed in animals exposed to control odors. Scale of colors in each area was arbitrarily chosen to make the amplitude of the variance in c-Fos counts across stimuli evident. **The Supplementary Table 2 is provided as a separate worksheet file.**
